# Selenium and Selenoproteins in Immune Mediated Thyroid Disorders

**DOI:** 10.3390/diagnostics8040070

**Published:** 2018-10-04

**Authors:** Liliana R. Santos, Celestino Neves, Miguel Melo, Paula Soares

**Affiliations:** 1 Institute of Molecular Pathology and Immunology of the University of Porto (IPATIMUP), 4200-135 Porto, Portugal; santos.lilianaribeiro@gmail.com (L.R.S.); jmiguelmelo@live.com.pt (M.M.); 2 Serviço de Medicina 2B—Hospital de Santa Maria/Centro Hospitalar Lisboa Norte, 1649-035 Lisboa, Portugal; 3 Department of Pathology, Faculty of Medicine, University of Porto, 4200-319 Porto, Portugal; 4 Endocrinology Service, Hospital Center São João, 4200-319 Porto, Portugal; mcelestinoneves@gmail.com; 5 Instituto de Investigação e Inovação em Saúde (i3S), Universidade do Porto, 4200-135 Porto, Portugal; 6 Department of Endocrinology, Diabetes and Metabolism, Centro Hospitalar e Universitário de Coimbra, 3000-075 Coimbra, Portugal; 7 Faculty of Medicine, University of Coimbra, 3004-504 Coimbra, Portugal

**Keywords:** selenium, selenoproteins, essential trace element, supplements, autoimmune thyroid diseases, Hashimoto’s thyroiditis, Graves’ disease

## Abstract

Selenium is an essential micronutrient that is required for the synthesis of selenocysteine-containing selenoproteins, processing a wide range of health effects. It is known that the thyroid is one of the tissues that contain more selenium. The “selenostasis” maintenance seems to contribute to the prevention of immune mediated thyroid disorders. Prospective, observational studies, randomized, controlled studies evaluating selenium supplementation, and review articles that are available in Medline and PubMed have undergone scrutiny. The differences concerning methodology and results variability have been analyzed. Several authors support the idea of a potential efficacy of selenium (mainly selenomethionine) supplementation in reducing antithyroperoxidase antibody levels and improve thyroid ultrasound features. In mild Graves’ orbitopathy, selenium supplementation has been associated with a decrease of the activity, as well as with quality of life improvement. Future research is necessary to clearly understand the selenium supplementation biologic effects while considering the basal selenium levels/biomarkers, selenoprotein gene polymorphisms that may be involved, underlying comorbidities and the major clinical outcomes.

## 1. Introduction

Normal thyroid function depends on many elements that are necessary for the synthesis and metabolism of thyroid hormones [1]. Selenium (Se) is an essential trace element (atomic number 34), discovered by the Swedish chemist Jons Jacob Berzelius in 1817 [2]. He named the new element “σελήνη—Selene” the goddess of the moon in ancient Greece. After 200 years of its first description, the role and the relevance of Se in human health start to be recognized [3]. In the past, Se was even considered a carcinogen, and now it is recognized as a vital nutrient. Schwarz and Foltz have contributed to the change on the conception of selenium. Schwarz was interested in understanding the liver necrosis that is found in laboratory rats when feeding on a diet containing torula yeast. After changing torula yeast to Saccharomyces yeast, the problem disappeared. Through this experience, Schwarz was able to show that torula was deficient in selenium and identified the first selenium-responsive disease [4]. The thyroid gland, in particular, is characterized by a high concentration of selenium, which is incorporated in several selenoproteins with key functions in the gland [5]. Not surprising selenium deficiency may impair thyroid function. In the scientific community, Se is considered as a topic of increasing interest. In this article, we will summarize the interactions between selenium, selenoproteins and the immune mediated disorders of the thyroid gland.

### 1.1. Selenium Intake, Biotransformation and Toxicity

Selenium is present in environment in organic compounds (selenomethionine and selenocysteine) and in inorganic compounds (selenite and selenate) [5]. Selenomethionine is found in vegetable sources and selenium yeast. The inorganic forms are present in the soils and are the main components of dietary supplements [6]. Dietary Se is essential in trace amounts and it is obtained through a wide variety of food sources, including grains, vegetables, seafood, meat, dairy products, and nuts [7]. A Belgian study states that the main sources of selenium are meat products (31%), fish (19%), pasta or rice (12%), and bread or cereals (11%) [8]. Most of the selenium is absorbed in the small intestine (50–80%). Selenium is mainly excreted by the kidneys (60%), followed by intestine (35%), and only 5% is excreted in sweat or saliva [9].

It is estimated that about 15% of the world population suffers from Se deficiency and the intake of selenium varies extremely worldwide. In Europe, dietary selenium intake is around 40 μg per day. In the United States of America (USA) the dietary selenium intake ranges from 93 μg per day in women to 134 μg per day in men [10]. Regarding gender, in the UK, the recommended daily selenium allowance is 75 μg/day for men and 60 μg/day for women [9]. The reasons for the world variability in intake include not only the selenium content in the soil, but also factors that determine the availability of selenium to the food chain (selenium type, soil pH, organic matter content, and ions) [11]. For example, selenium content in most parts of Europe is lower than in the United States and in average the selenium intake in Eastern Europe is lower than in Western Europe. For instance, in Finland the selenium content of the soil is extremely low, which resulted in a high incidence of selenium deficiency diseases. In order to increase the Se intake, the Ministry of Agriculture and Forestry in 1984, introduced a multinutrient fertilizer with sodium selenate. The plasma Se concentrations in healthy Finnish adults have been monitored regularly. In the 70s, the low dietary intake of Se, 25 μg/day was reflected in a plasma Se level of 0.63–0.76 μmol/L, which was among the lowest values that were reported in the world. With the supplementation of fertilizers with Se, the plasma selenium concentrations have increased by 70 percent, a level that is above the general plasma Se value in Europe, but is lower than that found in Canada or USA [12].

On the other hand, in the Amazonas region, there is a high Se intake. It is known that Brazil nuts (from the Brazil nut tree—*Bertholletia excelsa*) are the richest food source of selenium. In 2017, Silva Junior and collaborators verified that the median Se concentration in Brazil nuts ranged from 2.07 mg·kg^−1^ (in Mato Grosso state, Brazil) to 68.15 mg·kg^−1^ (in Amazonas state, Brazil). When the soils were analyzed the highest Se concentrations were observed in soil samples from the state of Amazonas. Therefore, Se accumulation in Brazil nuts is increased in soils with higher Se content [13].

China has areas of selenium deficiency affecting up to 105 million of people and areas where it is reported cases of Se excess [14]. Overt Se deficiency results in a condition called Keshan Disease (KD), which is an endemic cardiomyopathy occurring in low selenium areas of China. KD results in heart failure, cardiac enlargement, arrhythmias, and premature death. This condition has been associated with Se intake of 20 µg/day or less and it is known to be responsive to sodium selenite supplementation, although some authors consider that KD cannot be explained solely on low selenium status [15]. When describing selenium excess, Selenosis (a condition that can arise when selenium concentration exceeds 400 μg per day) should be also considered. Se toxicity symptoms are garlic breath, hair and nail loss, disorders of the nervous system, including paralysis, skin diseases, and poor dental health [11], and it was first described in parts of the population of the Hubei Province of the People’s Republic of China. Remounting from 1961 to 1964, the morbidity was almost 50% and its cause was determined to be due to selenium intoxication [16]. Nowadays, extreme toxicity of Se is not commonly found in humans. Miscalculated supplement formulations, accidental overdose, or intentional poisoning [17] have been reported, as is the case of 201 people that were affected by acute selenium toxicity associated with a misformulated supplement of sodium selenite. After the supplement suspension, the urine selenium concentration returned to normality in weeks [18].

Concerning the secondary effects of excessive selenium intake, in one of the few long-term studies, which included 1202 individuals, the authors found an increased risk of diabetes (hazard ratio 2.7) in patients taking selenium at 200 μg/day. These results should be taken with caution, since the diagnosis of diabetes was self-reported. Additionally, the study took place in an area with high basal selenium content [19].

### 1.2. Selenium and Selenoproteins

**Figure 1:**
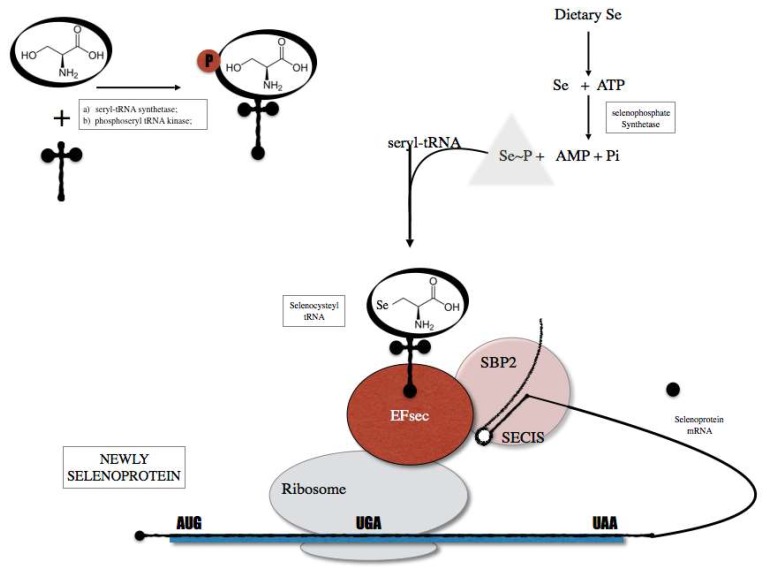
Selenoprotein biosynthesis pathway that is initiated by the charging of serine (Ser) onto a tRNA (tRNA Sec) in order to generate a Ser-tRNA Sec. The seryl-tRNA residue of Ser-tRNA Sec is then phosphorylated and converted to selenocysteyl-tRNA (Sec-tRNA sec). The Sec tRNA is used as Sec transfer into selenoproteins through the action of cis elements present in the selenoprotein mRNA and protein factors including SECIS binding protein 2 (SBO2) and Sec specific translational elongation factor (EF sec).

Selenium is inserted as selenocysteine (Sec). The biological effects of Se are mainly exerted through its incorporation into selenoproteins [20]. Dietary Se is incorporated within a complex selenoprotein biosynthesis pathway (Figure 1) present in all cell types. In humans, there are 25 identified genes encoding selenoproteins [21]. Mice lacking Sec tRNA^[Ser]Sec^ gene, required for the translation of selenoproteins, were embryonic lethal demonstrating that selenoprotein expression is essential for life [22]. Alterations in the expression of tRNA^[Ser]Sec^ results in changes on the expression of selenoproteins. Two different Sec tRNAs, that differ only on a single methylated base occurring on the 2′-O-hydroxyribosyl moiety at position 34 are expressed in different subclasses of selenoproteins, namely the housekeeping and the stress-related selenoproteins. Recently, Carlson et al. demonstrated new methods for the isolation and sequencing of selenocysteines tRNA [23], opening new windows for the study of selenoproteins proteasome.

### 1.3. Selenium and Thyroid Gland

**Figure 2:**
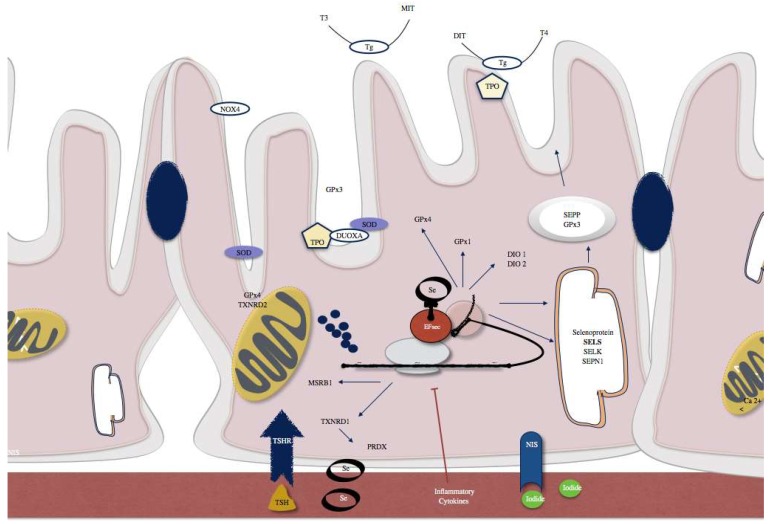
Examples of selenoproteins present in thyrocytes include SELENOP and glutathione peroxidase 3 (GPx3) that are actively secreted. SELENOS and SELENOK that have a role in quality control pathways within the endoplasmic reticulum (ER). Type 1 and 2 iodothyronine deiodinases (DIO1, DIO2), GPx1 and GPx4, methionine sulfoxide reductase B1 (MSRB1), selenophosphate synthetase 2 (SPS2), and thioredoxin reductase 1 (TXNRD1) are intracellular selenoenzymes. TXNRD2 and a GPx4 isozyme are localized in mitochondria. SBP2, which is a factor responsible for the selenoprotein translocation control.

The thyroid gland is characterized by a high concentration of selenium, and it is known that selenium and selenoproteins play an important role in thyroid function. In support of this hypothesis, low selenium levels have been found in the African area of Zaire where myxoedema is endemic, suggesting a role of this oligoelement. In 1987, Goyens and collaborators described myxoedematous endemic cretinism as a thyroid exhaustion atrophy [24]. In order to understand clearly the role of selenium, in a group of schoolchildren in the Northern Zaire with known deficiency of selenium and iodine, selenium supplementation resulted in a decrease in serum thyroxine and reverse triiodothyronine concentrations [25]. Since then, research on the implications of selenium on thyroid physiopathology was conducted. Most of the known selenoproteins are expressed in the thyroid gland (Table 1 and Figure 2). In 1973, glutathione peroxidase was the first selenoprotein characterized [26]. At the same time, it was documented the presence of two different enzymes that catalyze the conversion of thyroxine (T4) to triiodothyronine (T3): iodothyronine deiodinase type 1 and 2 (DOI 1 and 2). Later on, in 1991, it was proved that selenium was incorporated into iodothyronine deiodinases 1 (DOI 1) [27]. Therefore, selenium deficiency leads to a reduction on the expression and activity of theses enzymes, which results in an increase on T4 and a decrease on T3 levels. In accordance, experiments in rodents that have been fed with a low selenium diet showed increase in T4 levels and decrease in T3 levels [28]. Recently, Kawai and collaborators verified that children with severe selenium deficiency had high free T4 levels that were reduced with Se supplementation [29]. Among other well characterized selenoproteins, are the enzymes that are involved in the regulation of redox state and protection from oxidative damage, the thioredoxin reductases.

Biosynthesis of selenoproteins can be affected by germline mutations. *SECISBP2* gene encodes one of the essential components involved in co-translational insertion of selenocysteine (Sec) into selenoproteins. Mutation of *SECISBP2* is associated with a multisystem disorder and is reported by Schoenmakers and collaborators [30] when considering that the phenotype reflects tissue-specific effects caused by particular selenoprotein deficiencies and generalized antioxidant selenoenzyme deficiencies with excess cellular ROS [30]. Recently, it was proposed that patients with *SECISBP2* and *TRU-TCA1-1* defects manifest a multisystem disorder with a biochemical signature of abnormal thyroid function tests due to the impaired activity of deiodinase selenoenzymes, myopathy features that are linked to SEPN1 deficiency and phenotypes resulting from increased levels of reactive oxygen species attributable to lack of antioxidant selenoenzymes [31]. Dumitrescu and collaborators reported an inherited defect that was caused by a homozygous missense mutation of *SECISBP2* that disrupts thyroid hormone metabolism [32]. Another report detailed the *SECISBP2* gene mutation, R128X, which creates a premature stop codon in the molecule [33]. Recently, sequencing of DNA revealed a novel *SECISBP2* mutation in a Turkish boy that presented high T4, low T3, high rT3, and normal or slightly elevated TSH [34].

### 1.4. Selenium and Immune Mediated Thyroid Disorders

Graves’ disease (GD) and Hashimoto thyroiditis (HT) are the most common forms of autoimmune thyroid diseases (AITD). AITD affects more than 1–2% of the world population and it is diagnosed mostly in women between 30–50 years of age [35]. Graves’ disease is about one-tenth as common as HT and it tends to occur in younger individuals. Overall, a third of GD patients develop ophthalmic signs of Graves’ disease.

AITD pathogenesis is not yet clarified, but common and unique susceptibility genes have been proposed [36] in recent genome-wide association studies [37]. Recently, it was suggested that selenoproteins amount and activity are related to single nucleotide polymorphisms (SNPs) in selenoprotein genes, which can interfere with disease risk. Previous work that was published by our group suggested that there is a link between *SELENOS* (SEPS1) promoter genetic variation and Hashimoto’s thyroiditis (HT) risk. A total of 997 individuals comprising 481 HT patients and 516 unrelated controls were enrolled in the study. We found that individuals with *SELENOS*-105 GA and AA genotypes have a higher risk to develop HT (odds ratio (OR) 2.24, confidence interval (CI) 1.67–3.02, *p* < 5.0 × 10^−7^, versus, OR 2.08, CI 1.09–3.97, *p* = 0.0268, genotype, respectively) [38]; sexual dimorphism was also found in *SELENOS*-105GA genotypes distribution. Recently, Seale and collaborators highlighted the importance of considering sex differences in selenoproteins metabolism, expression, and action, which can be, in part, explained by endocrinological differences [39].

Reduced serum selenium concentrations (below 70 µg/L) are reported in patients with autoimmune thyroid disorders [40]. Several studies were undertaken in patients with AITD, especially in areas with low to borderline selenium content, aiming at understanding whether supplementation may have an impact on the evolution of thyroid immune mediated disease. The majority of the studies suggest that Se supplementation may decrease circulating thyroid autoantibodies, but the trials until now were heterogeneous in terms of number of patients, different forms of selenium supplements, duration of the supplementation, evaluation of thyroid by ultrasound, and serum selenium measurement. In Table 2, we presented a summary of the general characteristics and outcomes of selenium supplementation studies in patients with Hashimoto’s thyroiditis. Two groups were formed according to the type of supplement used (sodium selenite versus selenomethionine) and divided by doses or duration of treatment. In Table 2 is also shown whether the studies measured selenium levels at beginning or if the ultrasound features are checked during the trial.

Selenium supplementation in patients with AITD seems to modify the inflammatory and immune responses. The main proposed mechanisms include enhancing plasma GPX and TR activity and decreasing the toxic concentrations of hydrogen peroxide (H_2_O_2_) and lipid hydroperoxides [41,42].

Since 2002, several trials have investigated the effect of selenium supplementation. The first placebo-controlled clinical study with selenium in AITD was conducted in Bavaria/Germany, an area with known selenium deficiency. In this study, the effect of supplementing diet with 200 μg sodium selenite per day for 90 days was evaluated. Patients were divided into two groups: one group that was supplemented with sodium selenite and the other group that kept therapy with levothyroxine. At the end of the study, the concentration of anti-TPO antibodies decreased by 36% in the group that was treated with selenium (versus 10% in the placebo group). During this period, in 9 out 36 patients (25%) anti-TPO level was completely normalized and thyroid echogenicity also improved [43]. In an extension of Gartner study, one group of patients continued taking sodium selenite, which increased the reduction of anti-TPO to 43%, whereas the group that originally had received placebo was put on selenium and reached a reduction of 45% in anti-TPO antibodies levels [56].

Duntas and collaborators conducted a randomized, placebo-controlled trial, in which 65 patients with AITD were included [50]. In the group that were supplemented with selenomethionine, the level of TPOab decreased 46% after three months and 55.5% after six months, when compared to a decrease of only 21% and 27%, respectively, at three and six months in the group under isolated therapy with thyroxine [50].

Another study evaluated the effects of long-term (9 months) supplementation with variable doses of selenomethionine (100/200 μg per day) on autoimmune thyroiditis, particularly on the concentration of TPOab and TgAb. A total of 88 women with AITD, under thyroxine treatment were included in the study. The group supplemented during the nine months with 200 μg/day selenomethionine had a decrease in serum levels of TPOab until six months of treatment (26.6% at three months, 26.2% at six months) at nine months there was a decrease of only 3.6% in serum levels of TPOab. The authors proposed that doses of l-selenomethionine higher than 100 μg per day are necessary to suppress serum concentrations of TPOab in patients with AITD and to maximize glutathione peroxidase activities [47].

Nacamulli and collaborators, verified that supplementation with physiological doses of selenium (80 μg/day of sodium selenite) for 12 months reduces the echogenicity of the thyroid and TPOab and TgAb levels, but it does not modify TSH or FT4 [53].

Supplementation of Czech seniors with selenium rich yeast extract did not reduce TPO autoantibody concentrations [55]. Similarly, sodium selenite supplementation of Dutch patients with HT had no effect on TPO autoantibody concentrations, although serum selenium status did increase [46]. One study indicated that high (200 μg/day) but not moderate (100 μg/day) dosage is required to have a positive effect of supplementation. A low dose (80 μg/day) proved effective in another trial. No adverse effects were noted in these trials, and therefore, routine selenium supplementation in patients with Hashimoto thyroiditis has been discussed as a promising adjuvant treatment option [46].

Ferrari and collaborators recently published the beneficial effects of myo-inositol in association with selenomethionine in 21 patients [57]. The authors first showed an immune modulatory effect of myo-inositol in association with selenomethionine in patients with euthyroid AITD. After treatment, TSH levels significantly declined suggesting that the combined treatment can reduce the risk of progression to hypothyroidism in subjects with AITD [57].

Taking together the information collected in recently published systematic reviews, clinical trials, and meta-analysis, Se supplementation effectively reduces serum TPOab levels at 3, 6, and 12 months and serum TgAb at 12 months in LT4-treated populations. However, in LT4-treated patients, no significant correlation between the baseline serum Se and the decrease in serum TPOab level was demonstrated [58]. This meta-analysis also indicates that the formulation used is relevant, since a significant decrease in serum TPOab levels was seen in groups of patients receiving 200 μg selenomethionine, but not in those receiving 200 μg sodium selenite [58]. This can be related with the differential absorption of selenite that is approximately two-thirds of the absorption of selenomethionine. Still, in an additional meta-analysis present in the Cochrane library, the authors concluded that the available data is insufficient to support or refute the efficacy of Se supplementation in patients with HT [59]. It is important to note that, in most of the studies that focus on the relevance of selenium to thyroid disease, the authors did not measure selenium concentration prior to, during, and after supplementation. Furthermore, the most frequent primary outcome measurement was thyroid antibodies levels, so, at the present time, there is no recommendation for selenium supplementation in patients with AITD. The results to be obtained in an ongoing randomized controlled trial, the CATALYST trial (“The chronic autoimmune thyroiditis quality of life selenium trial”) that enrolled 472 patients with autoimmune thyroiditis treated with LT4, will be very important to settle the controversy. The primary aim of the trial is to study the effect of 12 months 200 μg selenium-enriched yeast supplementation versus placebo on thyroid-related quality of life. In this trial, at variance with other studies about this issue, plasma selenium concentrations will be measured periodically to assess selenium intake [60].

Postpartum Thyroiditis (PPT) is the most common and the most well characterized cause of thyroid dysfunctions in the postpartum period. Selenium supplementation has been suggested as a potential approach to the prevention and treatment of PPT. In Al-Kunani and collaborators’ study [61], women who suffered spontaneous abortion had lower selenium capillary levels than the group which had achieved term gestation and in the study by Negro and collaborators [62], supplementation of selenium was associated with significant reduction in anti-TPO antibody titer and significant improvement of the ultrasound thyroiditis pattern in pregnant women with autoimmune thyroiditis. This was accompanied by a significant reduction in the incidence of postpartum thyroid dysfunction and permanent hypothyroidism.

### 1.5. Selenium and Graves’ Disease

Several groups have analyzed the importance of selenium supplementation in patients with Graves’ disease. One of the first studies published in Graves’ disease evaluated the supplementation effect of a combination of antioxidants in a group of patients with Graves’ disease treated with methimazole. Patients who received supplementation with antioxidants in addition to therapy with methimazole attained more rapid biochemical and clinical remission than those who receive methimazole alone [63]. Wang and collaborators enrolled 41 patients with recurrent Graves’ disease who were under treatment with methimazole [64]. For six months, 21 patients were supplemented with sodium selenite (200 µg/day) in addition to methimazole vs. 20 patients without supplementation. The authors found a decreased of both, FT4 and FT3 at two months, in the selenium group when compared with the control group. A significantly lower level of TRAbs was detected in patients receiving selenium supplementation (2.4 IU/L vs. 5.6 IU/L, *p* = 0.04). At six months, a significantly higher percentage of patients with lower TRAbs levels was detected in the selenium group (19.0% vs. 0%, *p* = 0.016). These results suggest that selenium supplementation can enhance the effect of antithyroid drugs in patients with recurrent Graves’ disease (GD) [64].

The retrospective study performed by Wertenbruch in 2007 is pertinent [65]. The authors analyzed 83 patients with GD in an active form. Of that, 24 GD patients went into remission and the mean levels of anti-TSH antibodies (TRAbs levels) were significantly lower than in the relapse group (2.1 as compared to 8.6 IU/L; *p* < 0.0001). When remission and relapse group were compared, the serum Se levels in the remission group were higher (73.0 vs. 71.7 μg/L). The highest serum Se levels (>120 μg/L) were seen in the remission group, suggesting a role of Se levels in GD outcome [65]. At variance, Leo and collaborators found no effect of 166 µg/day adjuvant selenomethionine for three months in 30 Graves’ disease patients [66].

Graves’ orbitopathy (GO) is a major manifestation of autoimmune hyperthyroidism, which commonly affects patients with Graves’ disease. Marcocci and collaborators carried out a randomized, double-blind, placebo-controlled trial to determine the effect of selenium or pentoxifylline in 152 patients with mild Graves’ orbitopathy [67]. They found that treatment with selenium, but not with pentoxifylline, was associated with improved quality of life, less eye involvement, and delayed progression of Graves’ orbitopathy at six months. The patients were subsequently reassessed at 12 months (after 6 months without selenium, pentoxifylline, or placebo supplementation), and the results obtained in the first assessment were confirmed [67]. Although the evidence concerning selenium benefits in GO comes from this single randomized controlled study, a recommendation for its use in GO mild cases was incorporated into the recent guidelines from the European Group On Graves’ Orbitopathy (EUGOGO) [68].

In an Australian study incorporating around 200 patients, the authors reported significantly lower mean serum Se levels in patients with GO (1.10 ± 0.18 μm) when compared to GD patients without GO (1.19 ± 0.20 μm) (*p* = 0.001) [58]. In Dehina and collaborators’ study [69], serum Se and selenoprotein P (SELENOP) concentrations were determined in 84 consecutive patients with GO before treatment and compared to their clinical activity score (CAS), severity of eye changes (NOSPECS) status, and to the concentrations of TRAbs or the IGF1 receptor (IGF1R-aAB). The authors verified that the Se serum levels and SELENOP were associated, “indicating a suboptimal Se status of GO patients” [69]. The majority of GO patients from this German cohort had a relatively poor Se status ([Se] ± SD; 70.0 ± 23.8 µg/L), below the threshold needed for full expression of selenoproteins. However, the retrospective nature of the study is a limitation to establish conclusions on a potential causative role of Se on GD and/or GO risk [69].

The ongoing GRASS trial (GRAves’s disease Selenium Supplementation trial) that enrolled 492 patients with Graves’ hyperthyroidism can bring, in the future, more information in the issue [70]. The purpose of this study is to investigate selenium supplementation (200 μg/day of selenium-enriched yeast) in complement to the standard treatment with anti-thyroid drugs in patients with Graves’ hyperthyroidism during a long period of time (24 to 30 months). The outputs of the study are response to anti-thyroid drugs treatment, time for remission and duration of the remission, and quality of life during the first year of treatment [70].

## 2. Conclusions

Selenium supplementation is not yet recommended in international guidelines for treatment of AITD. Using a questionnaire study among Italian Endocrinologists, Negro, reported that selenium supplementation is currently used despite not being recommended in the guidelines. However, regarding Graves’ orbitopathy, the European Thyroid Association recommends a six-month trial period. Selenium supplementation in patients with Hashimoto’s thyroiditis with known selenium deficiency may be useful, even for those who are already being treated with levothyroxine, although further studies are needed to confirm this benefit. In patients with mild to moderate Graves’ orbitopathy, selenium supplementation seems to be beneficial and the organic formula (selenomethionine) seems to be more efficient than the inorganic formula. Additional studies will allow for the stratification of patients that are most likely to benefit from selenium supplementation.

## Figures and Tables

**Table 1:** Selenoproteins expressed in the thyroid and/or involved in the biosynthesis of thyroid hormone and protection of the thyroid gland (adapted from [5,14,20]).

Selenoproteins	Abbreviations	Function
Glutathione Peroxidase	Gpx	Catalyzes H_2_O_2_ reduction; Protects against oxidative stress
Cytosolic GPx1	cGPx1	Antioxidative defense; Type of reserve
Extracellular GPx	pGPx 3	Anti-inflammatory action
Phospholipid GPx	GPx4	Decreases phospholipid hydroperoxidases, moderates apoptosis
Iodothyronine deiodinase	DIO	Conversion of active thyroid hormone T3, reverse T3 and T2
Type I DI	DI-I	Conversion of T4 to T3
Type II DI	DI-II	Local production (intracellular) of T3 from T4
Type III DI	DI-III	Conversion of reverse T3 from T4 and T2 from T3
Thioredoxin reductase	TXNRD	Oxidoreductase activity having NADPH as a cofactor
Cytosolic TRx-1	TRx1	Regulates cellular redox level, cell development and proliferation
Mitochondrial TRx	TRx2	Regulates cell proliferation, tissue development
Various		
Selenoprotein P	SELENOP	Selenium transport, antioxidant defense
Selenoprotein N	SELENON	Degradation H_2_O_2_
Selenoprotein S	SELENOS	Quality control within the endoplasmic reticulum
Selenoprotein K	SELENOK	Quality control within the endoplasmic reticulum
Methionine sulfoxide reductase B1	MSRB1	Oxidative stress protection

The table shows different selenoproteins with known thyroid-related functions. T4—thyroxine; T3—triiodothyronine; T2—3,5-diiodo-l-thyronine; H_2_O_2_—Hydrogen peroxide.

**Table 2:** Summary of studies of selenium supplementation in patients with Hashimoto’s thyroiditis and corresponding outcomes.

Sample Size (F/M)	Supplementation Regimen	Duration of Treatment	Level of Se	TPOab Levels	Ultrasound Evaluation	Country	Reference
71 (71/0)	200 μg Na_2_SeO_3_ per day	90 days	√	Decreased by 36%	√	Germany	Gartner 2002 [43]
36 (36/0)	200 μg Na_2_SeO_3_ per day	90 days	×	No effect	×	Austria	Karanikas 2008 [44]
70 (45/25)	200 μg Na_2_SeO_3_ per day	3 months	√	No effect	×	Iran	Kachouei 2018 [45]
61 (NA)	200 μg Na_2_SeO_3_ per day	6 months	√	No effect	√	The Netherlands	Eskes 2014 [46]
88 (88/0)	200 μg SeMet per day	3 months	×	Decreased by 26%	×	Turkey	Turker 2006 [47]
86 (53/33)	200 μg SeMet per day	3 and 6 months	√	Decreased TgAb	√	Greece	Anastasikakis 2012 [48]
55 (50/5)	200 μg SeMet per day	3 months and 6 months	√	Decreased by 5% (3 m.) and 20% (6 m.)	√	Brazil	De Farias 2015 [49]
65 (56/9)	200 μg SeMet per day	6 months	√	Decreased by 56%	×	Greece	Duntas 2003 [50]
80 (80/0)	200 μg SeMet per day	6 months + 6 months	×	Decreased by 21%	×	Greece	Mazokopakis 2007 [51]
76 (76/0)	166 μg SeMet per day	6 months	×	No effect	√	Italy	Esposito 2016 [52]
88 (88/0)	100 μg SeMet per day	3 months	×	No effect	×	Turkey	Turker 2006 [47]
76 (65/11)	80 μg SeMet per day	6 months and 1 year	×	Decreased after 12 m.	√	Italy	Nacamulli 2010 [53]
60 (60/0)	80 μg SeMet vs. 160 μg SeMet per day	1 year	×	No effect	√	Italy	Pilli 2015 [54]
253 (NA)	100 μg yeast derived Se	1 year	×	No effect	×	Czech Republic	Kvicala 2009 [55]

F, female; M, male; NA, not available; Na_2_SeO_3_, sodium selenite; SeMet selenomethionine; TPOab; thyroid peroxidase antibody. × not done; √ evaluated.
